# Comparative Study of the Results of Operations in Patients with Tumor and Non-Tumor Obstructive Jaundice Who Received and Did Not Receive Antioxidant Therapy for the Correction of Endotoxemia, Glycolysis, and Oxidative Stress

**DOI:** 10.3390/antiox11061203

**Published:** 2022-06-20

**Authors:** Victor Stupin, Igor Abramov, Teymur Gahramanov, Alexey Kovalenko, Natalia Manturova, Petr Litvitskiy, Zalim Balkizov, Ekaterina Silina

**Affiliations:** 1 Department of Hospital Surgery No.1, N.I. Pirogov Russian National Research Medical University, 117997 Moscow, Russia; stvictor@bk.ru (V.S.); abramov-1961@mail.ru (I.A.); teymur_q@mail.ru (T.G.); manturovanatali@yandex.ru (N.M.); balkiz@mail.ru (Z.B.); 2 Chemical Analytical Department, Institute of Toxicology of the Federal Medical and Biological Agency of Russia, 192019 Saint Petersburg, Russia; kovalenko.alk@gmail.com; 3 Institute of Biodesign and Modeling of Complex Systems, I.M. Sechenov First Moscow State Medical University (Sechenov University), 119991 Moscow, Russia; lisovikp@mail.ru

**Keywords:** surgical jaundice, benign jaundice, malignant jaundice, oxidative stress, lipid peroxidation, biomarkers, hyperbilirubinemia, endotoxicosis, prognosis, antioxidant therapy, succinate

## Abstract

Purpose: To compare the results of surgical treatment and changes in biomarkers of cholestasis, endotoxicosis, cytolysis, lipid peroxidation, glycolysis disorders, and inflammation in patients with benign and malignant obstructive jaundice (OJ) in patients receiving and not receiving antioxidant pharmacotherapy (AOT). Patients and methods: The study included 113 patients (aged 21–90 years; 47 males and 66 females) who received surgical intervention for OJ due to non-malignant (71%) or malignant tumor (29%) etiologies. Patients were divided into two groups: Group I (*n* = 61) who did not receive AOT and Group II (*n* = 51) who received AOT (succinate-containing drug Reamberin) as part of detoxification infusion therapy. The surgical approach and scope of interventions in both groups were identical. Dynamic indicators of endotoxicosis, cholestasis, and cytolysis (total, direct, and indirect bilirubin, alanine aminotransferase [ALT], aspartate aminotransferase [AST], alkaline phosphatase [AP] and gamma-glutamyltransferase [GGT]), kidney function (urea), lipid peroxidation (malonic dialdehyde, MDA), inflammation (leukocytosis), and glycolysis disorders (lactate dehydrogenase (LDH), glucose) were evaluated. Results: Tumor jaundice, unlike non-tumor jaundice, persisted and was characterized by a more severe course, a higher level of hyperbilirubinemia, and lipid peroxidation. The prognostic value of the direct (and total) bilirubin, MDA, glycemia, and leukocytosis levels on the day of hospitalization, which increased significantly in severe jaundice and, especially, in deceased patients, was established. Decompression interventions significantly reduced levels of markers of liver failure, cytolysis, cholestasis, and lipid peroxidation on day 3 after decompression by 1.5–3 times from initial levels; this is better achieved in non-tumor OJ. However, 8 days after decompression, most patients did not normalize the parameters studied in both groups. AOT favorably influenced the dynamics (on day 8 after decompression) of total and direct bilirubin, ALT, AST, MDA, and leukocytosis in non-tumor jaundice, as well as the dynamics of direct bilirubin, AST, MDA, glucose, and LDH in tumor jaundice. Clinically, in the AOT group, a two-fold reduction in the operative and non-operative complications was recorded (from 23% to 11.5%), a reduction in the duration of biliary drainage by 30%, the length of stay in intensive care units was reduced by 5 days, and even hospital mortality decreased, especially in malignancy-induced OJ. Conclusion: A mechanism for the development of liver failure in OJ is oxidative stress with the appearance of enhanced lipid peroxidation and accompanied by hepatocyte necrosis. Inclusion of AOT in perioperative treatment in these patients improves treatment outcomes.

## 1. Introduction

The syndrome of obstructive jaundice (OJ), also known in the medical literature as surgical jaundice, is one of the five most common diseases that require urgent surgical treatment. The frequency of OJ is 18% or more among all surgical patients with biliary tract pathology, which is approximately 5 cases per 1000 individuals [1,2,3,4]. According to the etiopathogenesis, OJ is fundamentally divided into benign (non-tumor) and malignant (tumor). The main cause of benign OJ in adults is obstruction of the common bile duct or large duodenal papilla with calculus in gallstone disease; while malignant OJ mainly develops from tumor lesions of the pancreatic head, which block the free removal of bile into the duodenum [3,4,5,6]. Although the resulting OJ, both in both etiologies, is most often asymptomatic for the patient, the development of instrumental diagnostic methods allows the early identification of the cause of jaundice and determination of the type of surgical intervention required already at the first visit to the doctor. The use of modern surgical treatment, anesthesia, and recovery have undoubtedly contributed to the improvement of the results of treatment of patients with OJ [7,8,9,10]. Despite these successes, interventions performed over the past decade have not dramatically improved the outcomes of these patients. Postoperative mortality rates for OJ remain high and increase in severe patients and in complicated cases: postoperative mortality among patients with benign jaundice varies from 4% to 23%, and in patients with malignant tumor jaundice from 13% to 60% [4,11,12,13,14,15,16].

Clinically, mortality in patients with OJ is due to prolonged biliary obstruction with the development of necrobiotic and degenerative changes in liver tissue, and progressive endotoxemia against a background of existing general comorbidity [17,18]. Decompression interventions in the biliary tract are often performed against an unfavorable background due to the long disease course, and the existing methods of correction of hepatic metabolic disorders do not comprehensively consider all the pathophysiological mechanisms that lead to the development of liver failure [19]. In this regard, a central objective for the treatment of OJ is a pathogenetically supported method to restore the functional activity of liver tissue, based on the preservation of its metabolism and general correction of endotoxemia detoxification [20,21,22,23,24,25]. Therefore, greater attention has been paid to the study of pathophysiological processes that occur not only in the liver but also in general processes, that affect other organs and systems in individuals with OJ, which allows for the determination of optimal management of patients in the perioperative period and the practical implementation of personalized medicine.

In the first 3–5 days of OJ, the permeability of the hepatocyte membranes increases, which is accompanied by the release of indicator enzymes, and the excretion of bile by hepatocytes continues to create additional pressure within the duct system and disrupt blood circulation through liver tissue. Persistent pressure in the bile ducts leads to an increase in liver cell dysfunction, with an increase in the amount of unconjugated bilirubin in the blood. Hepatocyte necrosis is observed and therefore the activity of aminotransferases increases in the blood [4,26,27]. However, here the question arises as to why an acutely emerging OJ quickly causes acute liver and kidney failure, while patients with a slowly progressing OJ, regardless of its genesis, can survive for many months without critical lesions of these systems.

Due to the absence of bile acids in the intestine, lipase is not activated, protein digestion is disrupted, emulsification of fats, and absorption of fat-soluble vitamins is impaired. Deficiency of vitamin K1 leads to a decrease in the synthesis of prothrombin, which disrupts blood clotting. As a result of all these changes, the antitoxic function of the liver is altered, which is accompanied by endotoxemia syndrome. Microvascular thrombosis develops in the kidneys, the level of urea and creatinine increases in the blood, and violations of the antitoxic function of the liver increase. Toxic substances penetrate the blood–brain barrier with the development of hepatic encephalopathy [28,29]. Cholestasis has a damaging effect on both the tubular epithelium and hepatocytes. Bile components in high concentrations observed in OJ (hydrophobic bile acids, bilirubin, cholesterol) have a toxic effect on hepatocyte mitochondria, and directly or indirectly block the respiratory cycle and oxidation of fatty acids, leading to intracellular energy deficiency. The result is a further dysfunction of the hepatocyte with the release of liver enzymes into the bloodstream and the stimulation of lipid peroxidation processes, leading to rapid damage and cell death, or in the case of chronic process, transformation into carcinoma is possible [30,31].

We based this on the assumption that free radical processes arising under conditions of discoordination of metabolism in the cells of the body are general. Consequently, markers of oxidative stress and lipid peroxidation can thus be considered objective indicators of not only the severity of the disease course but also may define the phase of OJ and its favorable or unfavorable prognosis. Oxidative stress has an important role in the pathogenesis of various diseases of the gastrointestinal tract, bowel, and liver [21,22,32,33,34,35]. Therefore, correcting oxidative stress may reduce the intensity of lipid peroxidation processes and improve treatment outcomes. We tested this hypothesis in patients with OJ of different age groups and etiology, to identify levels of biochemical markers in the blood that increase depending on the intensity of free radical processes occurring in the body.

The purpose of the study was to evaluate the results of surgical treatment and the dynamics of endotoxicosis, cholestasis, cytolysis, lipid peroxidation, glycolysis, and inflammatory disorders in patients with benign and malignant OJ who received or did not receive succinate-based antioxidant pharmacotherapy.

## 2. Materials and Methods

### 2.1. Study Population

This retrospective comparative study included 113 patients with various tumor and non-tumor pathologies of the hepatopancreato-duodenal zone complicated by OJ was carried out. All patients were hospitalized in surgical and intensive care units at the University Surgical Clinics of the Pirogov Russian National Research Medical University, JSC Group of medical companies “Medsi” (Moscow, Russia), in the period 2018–2020. Inclusion criteria were: the presence of instrumentally confirmed OJ according to ultrasound, endoscopic examination, and/or computed tomography; adults over 18 years of both sexes; hyperbilirubinemia upon hospitalization; receiving endoscopic intervention for the treatment of jaundice (papillotomy, dilation of stenosis of the distal common bile duct, stenting choledoch) or video surgery, and if impossible, open methods (microcholecystostomy, cholecystectomy with drainage of the common bile duct).

The reasons for the development of the non-tumor jaundice were cholelithiasis and choledocholithiasis, chronic stenotic processes in the distal common bile duct, cholangitis, and acute pancreatitis. 

Pathologically, tumor jaundices were caused by adenocarcinomas. Tumors of the pancreas were represented by intraductal adenocarcinomas, the histological form of cancer of the major duodenal papilla was also adenocarcinoma, the tumor of the gallbladder was adenocarcinoma in all cases. Only patients without signs of metastasis were included in the analysis.

Most of the patients had comorbidities, but not in stages that could affect the results of treatment and the duration of hospitalization (i.e., patients with severe diabetes, hypertension, kidney disease, heart failure, etc., were not included in the study).

Surgical treatment was carried out according to the protocols adopted by the university clinic aimed at the earliest possible elimination of hypertension in the biliary tract with the implementation, if possible, of various types of minimally invasive decompression of the biliary tract and subsequent hepatotropic therapy. The resolution of cholestasis was carried out after the cause of OJ was established. Endoscopic papillosphincterotomy (EPST) with calculus removal was used more often (*n* = 57; 50.4%) and was performed only in patients with benign jaundice. If endoscopic clearance of the bile duct was not technically feasible, microcholecystostomy or gallbladder puncture (puncture cholecystostomy) was performed under ultrasound guidance (*n* = 17, 15%; including 11 (64.7%) patients with tumor jaundice). Nasobiliary drainage or stenting was performed in 22 (19.5%) patients, of which the majority (*n* = 14; 63.6%) were diagnosed with neoplastic OJ. Cholecystectomy, including drainage of the choledoch, was performed in 17 (15.0%) cases (9 (52.9%) with benign tumors and in 8 (47%) cases of tumor-induced OJ. In this study, we combined into one subgroup patients who underwent surgery by puncture cholecystostomy and cholecystectomy with drainage of the common bile duct as the cholecystostomy and cholecystectomy (CS and CE) group, since in all these cases there was a rapid discharge of bile with a decrease in pressure in the biliary tract. Such operations were performed in 34 (30.1%) patients (19 with neoplastic and 15 patients with non-neoplastic OJ).

At hospitalization, all patients underwent standard drug therapy, including intravenous infusion of third and fourth-generation cephalosporins, fluoroquinolones, antispasmodics, analgesics, and hepatoprotectors.

In addition, the analysis of the pharmacological treatment performed allowed the patients to be divided into two groups based on whether or not antioxidant therapy was performed. Antioxidant therapy (AOT) was prescribed randomly, depending on the preferences of the attending physician, the day of admission, the availability of the drug, or other circumstances. This study included only those patients who did not receive any AOT or were using as an infusion the detoxifying antioxidant meglumine sodium succinate (Reamberin^®^) at a dose of 400 mL per day by intravenous drip from the first day after the resolution of cholestasis for 5–8 days.

The composition of the medicinal antioxidant preparation Reamberin^®^: 1.5% infusion solution of meglumine sodium succinate, of which the ionic composition was sodium 147 mmol/L, potassium 4.02 mmol/L, magnesium 1.26 mmol/L, chlorides 109 mmol/L, succinates 46.0 mmol/L, meglumine 44.7 mmol/L. The drug Reamberin^®^ is used (main indication) as an antihypoxic and detoxifying agent for acute endogenous and exogenous intoxications of various etiologies in adults and children from 1 year of age. Contraindications for use: condition after a traumatic brain injury, accompanied by cerebral edema; acute renal failure; chronic kidney disease (stage 5, glomerular filtration rate less than 15 mL/min); pregnancy; breastfeeding period; individual intolerance.

### 2.2. Ethics

This study was approved by the Independent Ethics Committee JSC Group of medical companies “Medsi” (protocol No. 83 dated 28 Dec 2020; Moscow, Russia). This was a retrospective study. This study did not provide any additional intervention. During the study, personal data of patients were not disclosed. The inclusion of the patient in the study did not lead to a change in the general approach to treatment, regimens, and/or doses of the drugs used, which corresponded to the standards of medical care. All drugs that were prescribed to patients included in the study were registered within the territory of the Russian Federation. All patients signed an informed consent upon admission.

### 2.3. Methods

The assessment of the patients’ condition was carried out dynamically at three timepoints: at hospitalization (day 0), on day 3, and at days 8–9 after the elimination of the biliary obstruction.

Diagnostics included: general and biochemical blood tests, ultrasound examination of the organs of the gastropancreatoduodenal zone was performed using the Philips Epiq Elite (Philips, Andover, KS, USA), LOGIQ E9 (GE Healthcare, Chicago, IL, USA), Esaote Mylab Twice (Esaote, Genoa, Italy); endoscopic retrograde cholangiopancreatography (ERCP) was performed using video duodenoscopes TJF-Q190V (Olympus Medical, Japan) and Pentax ED-3430TK (Pentax Medical, Tokyo, Japan). Computed tomography (CT) was performed when indicated using a 256-slice Siemens SOMATOM Definition Edge computed tomography device (Siemens, Munich, Germany). Clinical assessment of severity during hospitalization was based on the SOFA scale.

Biochemical blood tests were performed using the biochemical analyzers Beckman Coulter AU480 (Beckman Coulter, Brea, CA, USA), Roche Cobas c 311 (Roche Diagnostics, Basel, Switzerland). Biochemical indicators of hepatic function included total bilirubin, direct and indirect bilirubin, indicators of alanine aminotransferase (ALT), aspartate aminotransferase (AST), alkaline phosphatase (AP), and gamma-glutamyl transferase (GGT).

Endotoxicosis was present in all patients. On admission to the hospital (day 0), the bilirubin level exceeded the upper limit of normal in all patients. For all patients admitted to hospital in serious condition, the level of hyperbilirubinemia was significantly higher than in patients of moderate severity (*p* < 0.001). 

Renal function was assessed by the biochemical blood urea index. Inflammation was assessed by the number of blood leukocytes. In addition, we studied glucose and lactate dehydrogenase (LDH) as indicators of glycolysis status and energy deficiency.

To assess the intensity of lipid peroxidation (LPO), we used the quantitative determination of malondialdehyde (MDA), which is a secondary product of LPO formed during the oxidative destruction of cell membrane lipids [36,37]. An increase in MDA, according to most studies, is an early sign of metabolic disorders in the body, even at the preclinical stage of various diseases, although some publication bias was not completely ruled out in meta-analyses [38,39,40]. 

The MDA titer was determined in plasma by its reaction with 2-thiobarbituric acid and the formation of a chromogen complex having an absorption maximum in the red region of the visible spectrum at a wavelength of 532 nm. The optical density was measured using a Unico 2800 spectrophotometer (United Products Instruments, Dayton, NJ, USA) at a wavelength of 532 nm. The plasma concentration of MDA was calculated by considering the molar extinction coefficient of the formed complex. The end result was expressed in micromoles per liter of plasma (or nmol/mL), according to the formula:

(1) $$C=\frac{D\times V^{p}\times 10^{6}}{\varepsilon }$$

where *D* is the optical density at a wavelength of 532 nm; *V* is the volume of the reaction mixture; *p* is the dilution factor; 10^6^ is the conversion factor in μM; Ɛ is the coefficient of molar extinction of trimetype complex MDA with 2-thiobarbituric acid (1.56 × 10^5^ M^−1^ cm^−1^).

Reference MDA values were obtained when examining healthy people: 1.5–3.9 nmol/mL; Me = 2.75 nmol/mL.

The clinical and laboratory effectiveness of AOT was judged by the length of stay of patients in intensive care units, by the frequency of complications, and mortality, as well as by indicators of cholestatic and cytolytic syndromes, indicating the functional state of the liver.

### 2.4. Statistical Analysis

All statistical analyses were performed using the statistical program Statistical Package for the Social Sciences SPSS 23.0 software (IBM Company, Armonk, NY, USA). Descriptive statistics of continuous quantitative indicators are presented as the mean (M) and standard deviation (±SD) with a normal distribution, as well as the median (Me), the interquartile range (IQR) for distributions other than normal. The IQR is reported including Tukey fences. The normality of the distribution was assessed by the Kolmogorov–Smirnov test.

The correlation analysis was carried out according to the methods of Pearson and Spearman. To compare two independent samples with non-normally distributed continuous data, the nonparametric Mann–Whitney test was used, while the Kruskal–Wallis test was used for multiple comparisons. To compare two dependent samples with non-normally distributed continuous data, the Wilcoxon test was used. Qualitative variables were compared using the chi-square test (analysis of contingency tables). Differences were considered statistically significant at *p*-values < 0.05.

## 3. Results

### 3.1. Characteristics of the Patients Included in the Study

The age of the patients included in this study ranged from 21 to 90 years (mean (median) 72 years with an interquartile range (58–80) years), of which 41.6% were males (*n* = 47) and 58.4% were females (*n* = 66). The etiologies for the development of OJ included diseases of the organs of the gastropancreatoduodenal zone due to neoplasm (*n* = 33; 29.2%) and non-neoplastic causes (*n* = 80; 70.8%). Non-neoplastic jaundice due to developmental reasons consisted mainly of patients with cholelithiasis and choledocholithiasis (*n* = 32; 30.1%). Chronic stenotic processes in the distal common bile duct were the second most frequent causes of benign OJ (*n* = 22; 19.5%), while cholangitis (*n* = 14; 12.4%) and acute pancreatitis (*n* = 10; 8.8%) were rarer causes.

**Figure 1:**
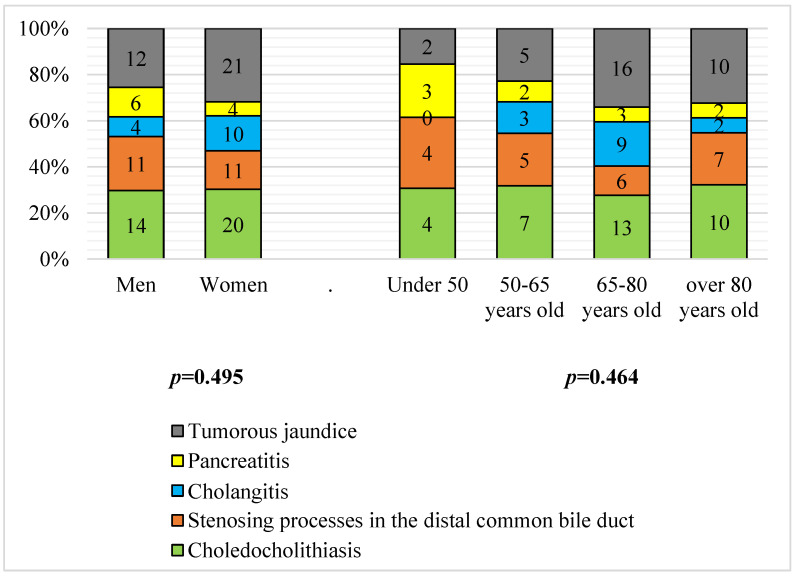
Distribution of males and females (*p* = 0.495) of different ages (*p* = 0.464) due to the development of OJ (*n* = 113); criterion X^2^.

Despite the absence of statistically significant differences in the development of OJ by sex and age, OJ in males (compared to women) was 2.1 times more frequent, OJ developed due to acute pancreatitis (12.8% versus 6.1%) and 1.4 times more often due to stenotic processes of the distal common bile duct (23.4% versus 16.7%); in women (compared to men), the OJ developed 1.8 times more frequently due to cholangitis (15.2% versus 8.5%). Tumor jaundice was registered in 25.5% of males and 31.8% of females (*p* > 0.05). Tumor jaundice was typical among older age groups over age 65 years, and in contrast, acute pancreatitis was more common in individuals aged under 50 years (Figure 1). Only patients without signs of metastasis were included in the analysis. Pathologically, tumor jaundices were caused by adenocarcinomas. Tumors of the pancreas were represented by intraductal adenocarcinomas, the histological form of cancer of the major duodenal papilla was also adenocarcinoma, the tumor of the gallbladder was adenocarcinoma in all cases.

Upon admission to the hospital, the severity of the patient’s condition, assessed using the Sepsis-related Organ Failure Assessment (SOFA) scale, ranged from 2 to 8 points. There were 63 (55.8%) patients of moderate severity (less than 4 points on the SOFA scale), 50 patients (44.2%) were severe (4 or more points); 40.7% of the patients (*n* = 46) were immediately admitted to the intensive care unit. The remaining patients were also transferred to intensive care units to prepare for the operation after laboratory and instrumental confirmation of the diagnosis of obstructive jaundice. Thus, all patients passed through the intensive care unit.

On hospitalization, all patients were diagnosed with hyperbilirubinemia. Depending on the total bilirubin levels, which were biochemically determined in blood plasma during hospitalization, the patients were distributed as follows: in 32.7% of cases, hyperbilirubinemia was <100 μmol/L, in 27.4% total blood bilirubin levels varied within the range of 100–150 μmol/L, and in 39.8% of patients, the level of total blood bilirubin was >150 μmol/L on day 1 of hospitalization. 

Depending on the presence or absence of treatment with AOT, the patients were divided into two groups statistically indistinguishable by common characteristics. Group I (comparison) included 61 patients (23 males and 38 females, mean age 67.2 ± 16.5 years) who received only standard conservative therapy, without AOT. Group II (main) consisted of 51 patients (24 males and 28 females, mean age 69.4 ± 14.6 years), who received AOT. As shown in Table 1, the groups were comparable in terms of sex, age, etiology of OJ, the duration of the clinically expressed OJ, and the severity at the time of hospitalization. The surgical approach and the number of surgical interventions in both groups of patients were identical (*p* > 0.05).

### 3.2. Severity and Duration of Jaundice according to Etiology

A significant difference in the severity of the OJ and its etiology was determined (*p* < 0.01). The majority (66.7%) of patients presented a severe condition during hospitalization with tumor jaundice (22 patients with severe tumors, 11 were in a state of moderate severity), as well as with cholangitis (9 severe and 5 moderate; 64.3%). In acute pancreatitis, 40% of patients were hospitalized with a serious condition. Patients with gallstones with choledocholithiasis and with distal stenoses of the common bile duct were more frequently (in 70.6% and 77.3% of cases) admitted to hospital in a state of moderate severity.

**Figure 2:**
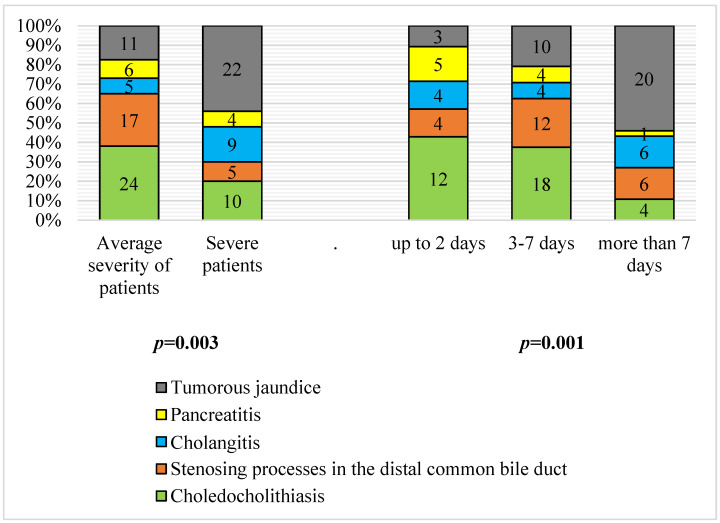
Distribution of patients with different causes of obstructive jaundice by severity (*p* = 0.003) and duration (*p* = 0.001); criterion X^2^ (*n* = 113).

The duration of cholestasis at the time of admission to the clinic ranged from several hours to 2 months, while 24.8% of the patients (according to the patients and/or their relatives) were admitted to the clinic within the first 2 days after the onset of pain in the right epigastric region and symptoms of jaundice; 42.5% of the patients were hospitalized within 3–7 days after the onset of jaundice. In 32.7% of patients, jaundice manifested clinically for more than a week, which is mainly characteristic of the tumor genesis of OJ. Early hospitalization is typical for acute pancreatitis (50% of patients were hospitalized on the first day) and cholelithiasis and choledocholithiasis (42.9% of patients with this pathology were hospitalized less than 48 h after clinically manifested jaundice). A statistically significant difference was determined when compiling the contingency tables for the etiology and duration of cholestasis (*p* < 0.01) (Figure 2).

**Figure 3:**
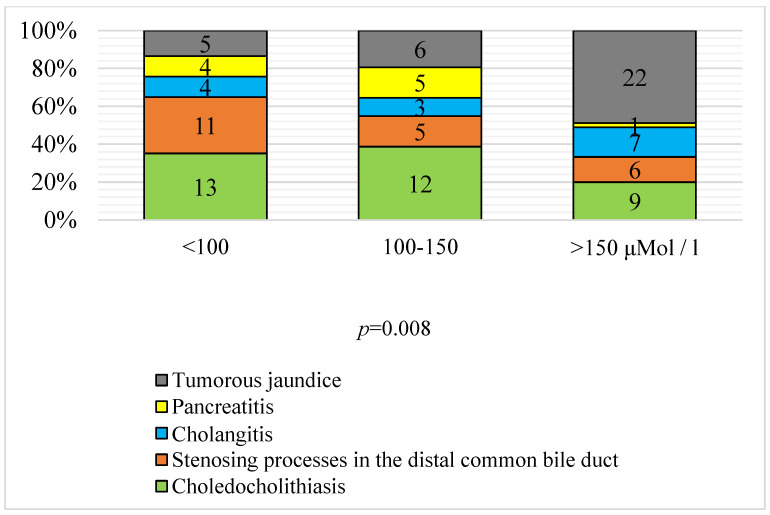
Distribution of patients with different causes of obstructive jaundice stratified by the level of hyperbilirubinemia after hospitalization (*p* = 0.008); criterion X2 (*n* = 113).

A statistical difference between the level of hyperbilirubinemia and the causes of OJ development was determined. The highest level of hyperbilirubinemia was characteristic of tumor jaundice (66.7% of neoplastic jaundice was accompanied by hyperbilirubinemia above 150 μM/L) and cholangitis (50%). Acute pancreatitis, choledocholithiasis, and chronic stenoses of the distal common bile duct were characterized by a lower level of hyperbilirubinemia (Figure 3).

Thus, tumor jaundice persisted for a long time and was distinguished by a more severe course and a higher level of hyperbilirubinemia.

### 3.3. Predictive Value of the Laboratory Parameters during Hospitalization

Despite ongoing surgical and pharmacological treatment, 13 (11.5%) patients with OJ died in hospital. Comparative analysis of indicators studied on day 0 during hospitalization in deceased and subsequently discharged patients according to the Mann–Whitney criterion established the most significant difference in indicators included direct bilirubin levels (2.36 times higher in deceased compared to discharged patients, *p* = 0.001), total bilirubin levels (1.75 times higher in deceased, *p* = 0.002), MDA levels (1.20 times higher in deceased patients, *p* = 0.027), glucose levels (1.20 times higher in deceased patients, *p* = 0.033), and the number of blood leukocytes (1.50 times higher in deceased patients, *p* = 0.043). Considering that 13 cases is a small sample size, we analyzed the same indicators in patients hospitalized with a serious condition (*n* = 50) and in a state of moderate severity (*n* = 63). The results of the combination Table 2 confirmed that hospital mortality was 7 times higher among patients hospitalized in a serious condition (22.0%) than in non-severe patients (3.2%; *p* = 0.002).

Therefore, a parallel analysis of identified prognostic markers is appropriate for patients with varying severity at the time of hospitalization. During the course of the study, patterns were obtained that indicated a unidirectional increase in prognostic markers, which increased regularly in proportion to the severity of the patients.

**Figure 4:**
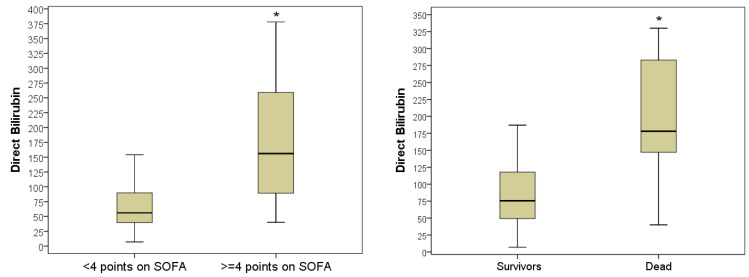
Indicator of direct bilirubin in hospitalization of severe and non-severe patients, as well as in favorable and unfavorable hospital outcomes (*—difference between the two groups according to the Mann–Whitney criterion at *p* < 0.05).

Therefore, the total bilirubin indicator in severe patients (≥4 points on the SOFA scale during hospitalization) varied between 47–378 μmol/L (5–95% percentile) and averaged 156 μmol/L (IQR, (89–259) μmol/L). This was on average 2.79 times higher than in non-severe patients, whose direct bilirubin level on the 1st day of hospitalization varied between 1–5138 μmol/L and averaged 56 (IQR, 39–89) μmol/L (*p* = 0.001). In patients who died in hospital, the level of direct bilirubin was the highest and averaged 178 (IQR, 14–283) μmol/L, in discharged patients, the level of direct bilirubin during hospitalization was lower by 100 μmol/L at a mean of 75.5 (IQR, 49.3–117.7) μmol/L (*p* = 0.001) (Figure 4).

**Figure 5:**
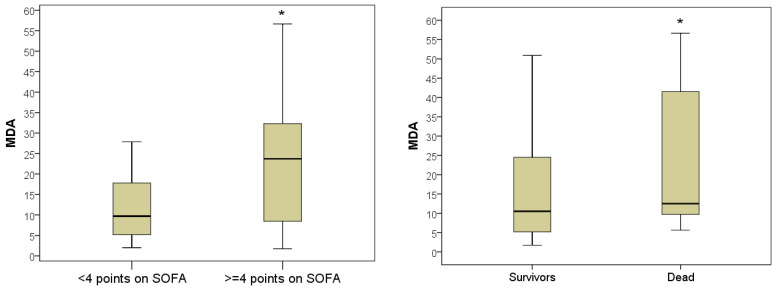
Malondialdehyde levels in blood plasma during hospitalization of severe and non-severe patients, and association with favorable and unfavorable hospital outcome (*—the difference between the two groups according to the Mann–Whitney criterion at *p* < 0.05).

Plasma MDA levels in severe patients varied from 2–52 nM/mL (5–95% percentile) and averaged 23.7 nM/mL with an IQR of (8.5–32.2) nM/mL. This was on average 2.44 times more than in non-severe patients, whose plasma MDA level on the day of hospitalization reached 27.9 nM/mL and averaged Me = 9.7 [IQR, 5.2–17.8] nM/mL (*p* = 0.003). In 100% of the deceased patients, the levels of MDA during hospitalization exceeded the upper limit of normal, varying in the range of 5.6–56.7 nM/mL and averaging 12.6 (IQR 9.7–41.5) nM/mL. In discharged patients, the MDA indicator was significantly lower and was 10.5 [IQR 5.2–24.5] nMol/mL (*p* = 0.027) (Figure 5).

**Figure 6:**
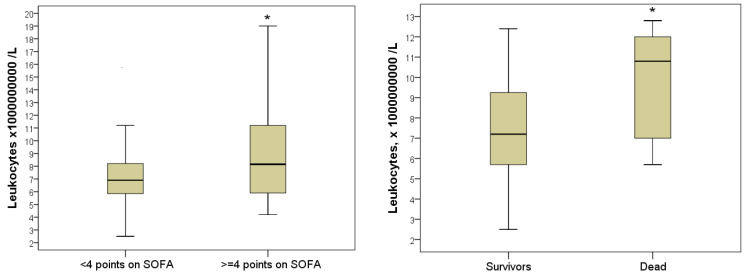
The number of blood leukocytes in hospitalization of severe and mild patients and in patients with favorable and unfavorable hospital outcome (*—the difference between the two groups according to the Mann–Whitney criterion at *p* < 0.05).

The number of leukocytes in severe patients during hospitalization was 8.2 [IQR 5.9–11.2] × 10^9^/L, which was on average 2.1 times higher than in non-severe patients (6.8 [5.8: 8, 2] × 10^9^/L; *p* = 0.049). The highest number of leukocytes during hospitalization was in those with a lethal outcome, on average 10.8 (IQR 7.0–12.1) × 10^9^/L, this was 1.5 times higher than in the surviving patients (Me = 7.2 [IQR 5.7– 9.9] ×109/L; *p* = 0.043). Leukocytosis greater than 10 × 10^9^/L was registered in 7 (53.8%) deceased patients (Figure 6).

**Figure 7:**
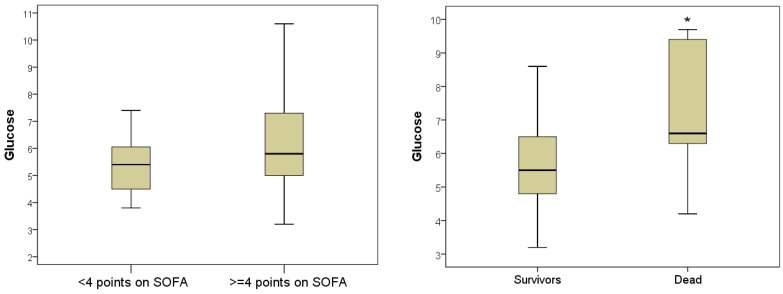
Blood glucose index during hospitalization in severe and non-severe patients and in patients with favorable and unfavorable hospital outcome (*—the difference between the two groups according to the Mann–Whitney criterion at *p* < 0.05).

The level of hyperglycemia has also been demonstrated to be predictive of outcome. Despite the absence of a statistically significant difference in glycemia level in severe (Me = 5.8 [IQR 5.0–7.3] mmol/L) and non-severe patients (Me = 5.3 [IQR 4.5: 6.3] mmol/L; *p* = 0.094), nevertheless, there was a trend for a more pronounced increase in this indicator in severe jaundice. Hyperglycemia ≥ 5.8 mmol/L was diagnosed in half of the severe patients. The comparative analysis of blood glucose levels in different hospital outcomes established a significant difference in the indicator. In those who died during hospitalization, the glycemic level averaged 6.6 mmol/L with an IQR of 6.3–9.4 mmol/L; in patients discharged patients, on average, the blood glucose level was 1.2 times lower (Me = 5.5 [IQR 4.8–6.5] mmol/L; *p* = 0.033). Hyperglycemia greater than 6.0 mmol/L was registered in 10 (76.9%) patients who died during hospitalization (Figure 7).

Therefore, the differences in laboratory parameters identified in patients with different hospital jaundice outcomes are consistent with the data in the analysis of severe and mild OJ patients. This allows us to conclude about the value of markers such as direct (and total) bilirubin, MDA, glycemia, and leukocytosis, which may act as early prognostic markers that allow to predict outcomes from the day of hospitalization and allow for promptly adjusting treatment.

The data obtained strongly suggest the mutually potentiating effects of hepatotoxicity, inflammation, lipid peroxidation, and glycolysis disorders in the pathogenesis of OJ. Therefore, correcting these disorders may improve treatment outcomes.

### 3.4. Influence of Surgical Intervention and Antioxidant Therapy on Laboratory Markers of Severity of Tumor and Non-Tumor Obstructive Jaundice 

Analysis of the dynamics of biochemical markers of liver function (i.e., ALT, AST, total bilirubin, direct and indirect bilirubin, alkaline phosphatase, and GGT) in patients with OJ of various origins and severity revealed a prolonged increase in their titer with worsening OJ. The most pronounced regression of biochemical parameters was observed in the first days after decompression; however, after 8 days of resolution of cholestasis, all parameters studied exceeded the normal values.

The inclusion of AOT in the treatment of patients with OJ of various origins and severity led to a more pronounced regression of the cytolysis and cholestasis indices, ahead of the comparison group and standard therapy (Table 3).

Decompression surgery significantly reduced the levels of markers of liver failure. On the third day after decompression, the level of blood bilirubin levels decreased 2–3-fold from initial values, and to a greater extent for direct bilirubin levels. Additional prescription of AOT led to a more significant regression of hyperbilirubinemia. On the third day after decompression, the level of direct bilirubin was on average 2.2 times higher in the comparison group than in the AOT group (on average 44.1 mmol/L versus 19.7 mmol/L, *p* < 0.05), and by 8 days it was 2.5 times higher (25.8 mmol/L versus 10.5 mmol/L, *p* < 0.05). There were no differences in the dynamics of the indirect bilirubin indicator in the study groups.

By analyzing the effects of AOT on the treatment outcomes of patients with neoplastic and non-neoplastic-induced jaundice, the following statistically significant intergroup differences were found. The level of total bilirubin was statistically different only in cases of non-neoplastic jaundice on day 8 (1.2 times lower on average in the AOT group, *p* < 0.05). Despite the fact that the level of total bilirubin in the comparison group with tumor jaundice was 1.9 times higher than in the AOT group, no statistically significant differences were found, which may however be associated with the smaller number of patients in these subgroups.

**Figure 8:**
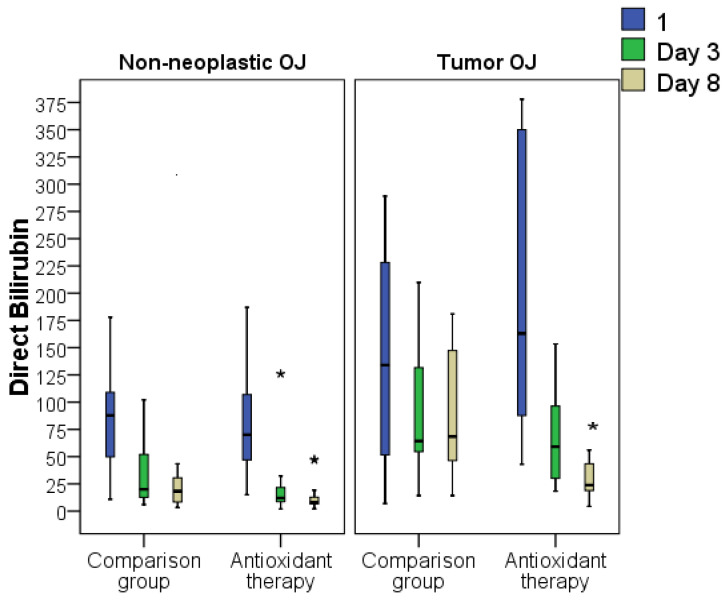
Dynamics of the direct bilirubin indicator in tumor and non-tumor jaundice in patients who received and did not receive antioxidant therapy (* difference between the comparison groups and AOT at *p* < 0.05). Abbreviations: OJ, obstructive jaundice.

The level of direct bilirubin in non-neoplastic OJ was significantly higher in the comparison group, by an average of 1.7 times on day 3 after decompression (*p* < 0.05), and 2.2 times on day 8 after decompression (*p* < 0.05). The level of direct bilirubin in tumor OJ differed in the groups only on day 8 after decompression, on average 2.9 times (*p* < 0.05) (Figure 8).

Analysis of the indirect bilirubin indicator did not reveal any intergroup differences in tumor or non-tumor OJ, although it tended to show a stronger association with the indicator when following AOT in patients with tumors.

The ALT indicator was significantly lower in the AOT group on day 8 after decompression (on average, 1.4 times, *p* < 0.05). A more significant effect of AOT in terms of AST was determined, which was, on average, more in the comparison group on day 3 (1.2 times, *p* < 0.05) and on day 8 (1.62 times, *p* < 0.05) after decompression. However, a differentiated analysis of these indicators in patients with tumor and non-tumor OJ revealed differences only on day 8 and only in the AST indicator, which was higher in the comparison group relative to the AOT group on average by 1.8 times in patients with non-tumor jaundice (*p* < 0.05) and 1.5 times in patients with tumor OJ (*p* < 0.05). A moderate increase in the activity of the intracellular enzyme AST is usually detected in liver cirrhosis, OJ, metastases, acute pancreatitis, or surgery. A significant increase in AST activity is observed with necrosis or damage to the lipid cell membrane of hepatocytes. Comparative analysis of the dynamics of indicators of AP and GGT did not reveal any differences between the groups. This is not surprising, since these enzymes are increased to a greater extent in cholestasis or severe acute alcohol poisoning and have a weaker correlation with necrotic death of hepatocytes. Therefore, the use of AOT showed a beneficial effect on the dynamics of bilirubin and hepatic enzymes in OJ of different etiologies.

**Figure 9:**
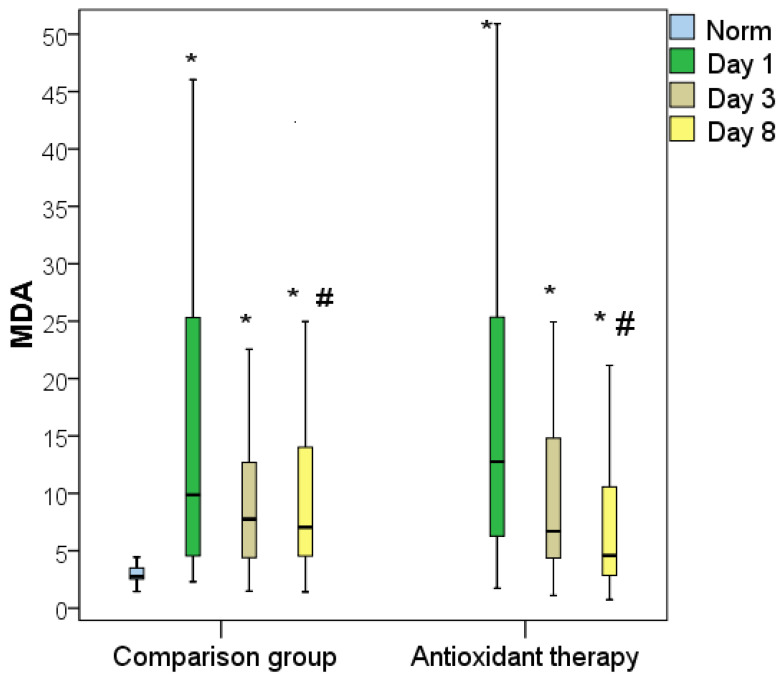
Dynamics of the plasma blood MDA index in obstructive jaundice in both groups (*—difference from the normal; #—difference between groups at *p* < 0.05, Mann–Whitney test).

Analysis of the dynamics of the MDA index revealed that the treatment of jaundice did not lead to a complete and rapid normalization of the MDA level. Despite the significant regression of this indicator after the surgical intervention in both groups, the MDA level significantly exceeded normal values even 8 days after decompression. The use of AOT as part of basic therapy was accompanied by normalization of the MDA level by 8 days of decompression in 40% of patients (15% in the comparison group). On the third day after surgical decompression, the mean concentration of MDA in the comparison group was 7.8 nmol/mL (IQR, 4.4–12.7 nmol/mL), which had decreased relative to the day of hospitalization by an average of 1.27 times. In the AOT group, the regression of MDA on day 3 was more pronounced, by up to 6.7 nmol/mL (IQR, 4.3–14.8 nmol/mL). Despite the absence of a statistical difference in the MDA index on day 3 after decompression in both groups, on average, the MDA level in the AOT group was higher on day 0 by 1.89 times than on day 3 (*p* < 0.05). That is, the use of AOT promoted a more pronounced regression of lipid peroxidation processes already 3 days after surgical decompression. On the eighth day after decompression, the average concentration of MDA in the comparison group was 7.1 nmol/mL (IQR, 4.5:14.0 nmol/mL). This corresponded to the MDA level on day 3 in the comparison group and was significantly 1.54 times higher than in the AOT group (*p* < 0.05). Furthermore, in the AOT group, the MDA level decreased significantly on day 8 compared to day 3 by an average of 1.45 times to 4.6 (IQR, 2.8–10.6 nmol/mL, *p* < 0.05) (Figure 9).

**Figure 10:**
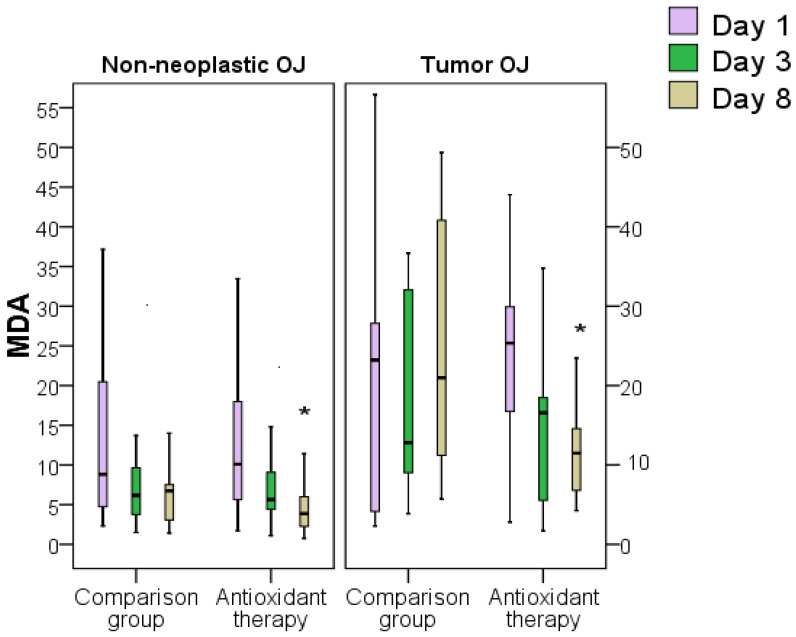
Dynamics of the blood plasma MDA index in obstructive jaundice of tumor and non-tumor genesis in both groups (*—difference between groups at *p* < 0.05, Mann–Whitney test). Abbreviations: OJ, obstructive jaundice.

When analyzing the MDA index in patients with OJ of tumor and non-tumor etiologies, there were no differences between the groups on day 3 after decompression, despite the tendency to achieve better dynamics in the AOT group. On the eighth day after the surgical intervention, the MDA level was significantly higher in the comparison group both in non-neoplastic jaundice 1.7 times (*p* < 0.05), and in tumor OJ 1.8 times (*p* < 0.05). demonstrating the effectiveness of a succinate antioxidant in detoxification and preservation of cell viability (Figure 10).

The use of AOT had a favorable effect on the dynamics of the biochemical marker of renal function, blood urea. On day 3 after surgical decompression, the difference in this indicator between the two groups was 1.2 times (on average, 5.6 [IQR, 3.8–9.1] mol/L in the standard therapy group and 4.7 [IQR 3.7: 6.3] mmol/L in the AOT-treated group; *p* < 0.05). On the eighth day after decompression, the urea level was also significantly higher in the comparison group (on average 5.3 [IQR, 4.6–9.0] mmol/L in the standard therapy group and 5.0 [IQR, 4.1–6.8] mmol/L in the group AOT; *p* < 0.05). At the same time, in 35% of the patients in the comparison group on the eighth day, the urea titer still exceeded the upper limit of normal, in the AOT-treated group, such cases were less than 10%. When analyzing the blood urea index in tumor and non-tumor etiologies of OJ, a significant difference was found supporting the benefit of AOT in tumor jaundice on day 8 (on average, 7.1 [IQR, 4.5–15.0] mmol/L in the standard therapy group and 5.8 [IQR, 4.3–6.5] mmol/L in the AOT-treated group; *p* < 0.05).

The dynamics of a marker of inflammation (leukocytosis) also established the benefits of AOT. On the third day after decompression in the standard therapy group, the number of leukocytes was on average 1.13 times higher than in the AOT group (on average 7.6 × 10^3^ versus 6.7 × 10^3^ leukocytes). On the eighth day after the operation, this difference increased to 1.29 times (the number of leukocytes in the comparison group on the eighth day averaged 8.1 × 10^3^ (IQR, 5.7–12.2) and in the AOT group 6.3 × 10^3^ (IQR, 5–9 × 10^3^); *p* < 0.05). The differential analysis of the number of leukocytes with different OJ etiologies showed the advantages of the addition of AOT only in non-neoplastic jaundice (on day 8, the difference from the comparison group averaged 1.35 times, *p* < 0.05) (Table 4).

**Figure 11:**
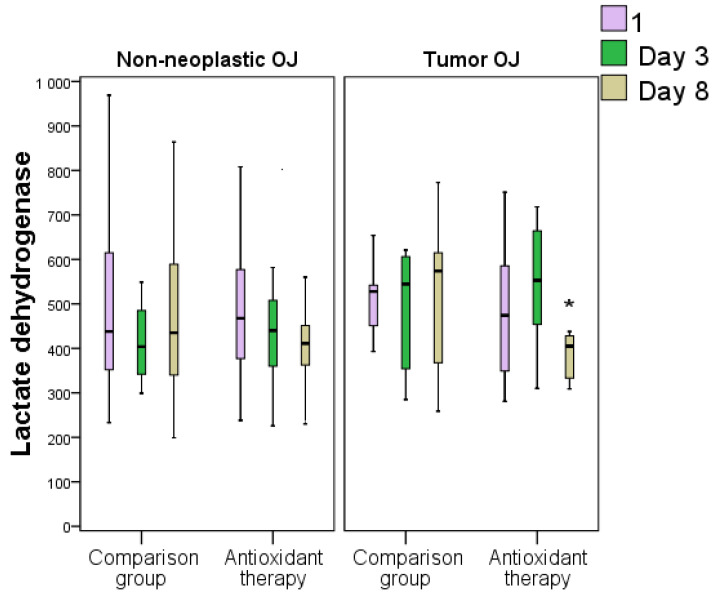
Dynamics of lactate dehydrogenase levels in blood plasma in obstructive jaundice of various origins following antioxidant therapy (*—difference between groups at *p* < 0.05, Mann–Whitney test). Abbreviations: OJ, obstructive jaundice.

Despite our hypothesis that AOT with succinates could influence glycolysis, the analysis of glucose and LDH levels established a significant difference on day 8 only in patients with OJ induced by tumors, the most severe jaundice etiology (in the AOT group, LDH was 1.33 times less on average and glucose was 1.13 times lower, *p* < 0.05). There were no differences between the non-neoplastic OJ groups in terms of changes in LDH and glucose indicators (Table 5, Figure 11).

### 3.5. Influence of Antioxidant Therapy on Treatment Results and Hospital Outcome of Obstructive Jaundice of Tumor and Non-Tumor Origin

In the patients receiving AOT, with the objective of normalization of laboratory parameters, an improvement in the clinical picture of the disease and a reduction in the number of complications were observed. In the patients who also received AOT, the incidence of all complications (operative and non-operative) was 11.5% (*n* = 6, all 6 patients had a non-neoplastic OJ), while in the comparison group, the incidence of all complications was two-fold higher and was 23.0% (*n* = 14, including 10 patients with OJ of non-tumor origin and 4 patients with OJ of tumor origin.

There was a decrease in the duration of biliary tract drainage of 1.45 times (by 30.79%) (*p* < 0.001), with the duration of preoperative bile diversion from the moment of decompression to normalization of the intoxication indices in the comparison group of 12.7 ± 2.1 days, and in the main group 8.8 ± 1.9 days (*p* < 0.001). When prescribing AOT, a significant reduction in the length of stay of patients in intensive care units was recorded by an average of 5 days (*p* < 0.001). The duration of stay in the ICU in the comparison group was 11.7 ± 10.1 days (Me = 7 [IQR, 3: 18] days), and in the main group, this decreased to 2.8 ± 2.5 (Me = 2 [IQR, 1–4]) days (*p* < 0.001).

**Figure 12:**
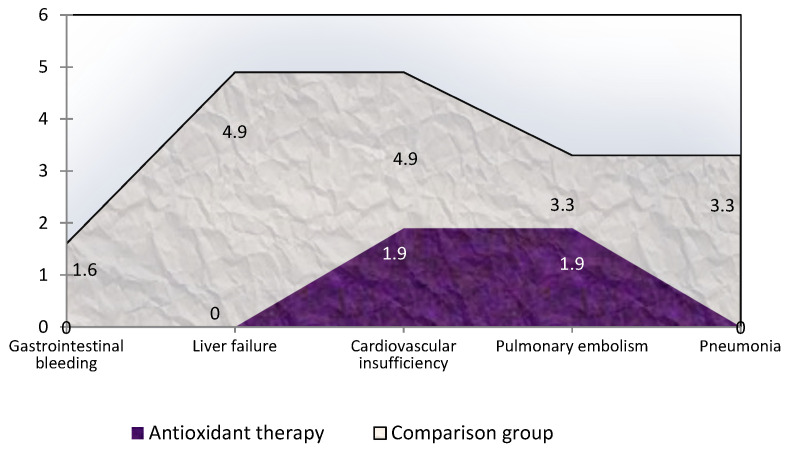
Frequency (%) and causes of hospital mortality in patients with obstructive jaundice who received and did not receive antioxidant therapy.

The comparative analysis of mortality in the two study groups of patients revealed a significant decrease in hospital mortality in patients treated with AOT. In patients who received AOT in the hospital, 2 (3.8%) patients died, and both cases had a non-neoplastic-induced OJ (the cause of jaundice was cholelithiasis, choledocholithiasis). The fatal outcome of one patient in this group was due to progressive cardiovascular insufficiency, and in the second patient pulmonary embolism. Of the patients in the comparison group, hospital mortality was 4.7 times higher and amounted to 18.0% (11 patients, including 6 (14.0%) of patients with non-neoplastic-induced OJ and 5 (27.8%) tumor-induced OJ). The reasons for the development of non-neoplastic jaundice in the deceased patients of the comparison group were cholelithiasis choledocholithiasis (*n* = 3), chronic stenotic processes of the choledochal (*n* = 2), and cholangitis (*n* = 1). The main cause of death in the 3 patients in the comparison group was progressive hepato-renal insufficiency in 3 patients, cardiovascular insufficiency in 2, pulmonary embolism in 1 patient, gastrointestinal bleeding, and in 2 patients bilateral pneumonia and/or pulmonary edema (Figure 12).

A significant decrease in hospital mortality was determined in general. This was attributed to the mortality of patients with inoperable neoplastic jaundice in the comparison group, while in the AOT group all patients with neoplastic jaundice survived and were discharged from the hospital. There were no statistically significant differences in hospital mortality among patients with non-neoplastic-induced OJ between the groups, despite the upward trend in the survival rate from 86% in the comparison group to 94.6% in the AOT-treated group (Table 6).

## 4. Discussion

Surgical resolution of OJ continues to be discussed with regard to the optimal timing of the surgical intervention, its nature, and the pharmacological management of patients in the preoperative and postoperative period [19,20,41]. Herein, we present an analysis that examined this issue from the standpoint of the pathophysiology of the process and obtained new scientific data, which, may reduce the number of complications in this syndrome and the degree of postoperative mortality. Improving treatment results of patients with OJ continues to be the focus of surgeons’ attention, both due to the increasing number of such patients and due to the persistence of a rather high mortality rate. Thus, in the present study, we analyzed the treatment outcomes of patients with surgical jaundice from the standpoint of the dynamic characteristics of the free radical processes involved. The relationship between free radical processes indicators and the severity of OJ was confirmed not only by our study but also by other studies conducted in different countries [21,22,34,35,36,42,43,44,45,46,47]. In animal models and in patients with OC, a marked increase in plasma levels of oxidative stress markers was recorded, while the level of antiperoxidase activity paradoxically decreased. We observed similar effects on parameters characterizing the free radical processes, and this increase was correlated with the severity of the patient.

Simple and logical decisions by the surgeon are not always correct from the standpoint of biological processes. For example, the rapid surgical resolution of cholestasis, which is accompanied by a sharp decrease in pressure in the biliary tract, worsened oxidative stress in patients with OJ and aggravated the course of liver and kidney failure. Of course, operational stress and anesthesia also contributed to this. However, the proportion of problems introduced by these factors, due to the development of minimally invasive technologies and modern anesthesia, is relatively low. The issue of the need for preoperative endoscopic or minimally invasive unloading of the biliary tract remains debatable. Although the development of modern technologies will allow surgeons to recommend this approach for benign, and therefore relatively “short” episodes of jaundice, such unanimity may not be achieved for the treatment of patients with tumors of the pancreatic head. Even reviews of the literature do not provide a clear answer to the question. Some studies demonstrate the effectiveness of preoperative biliary drainage in malignancy-induced OJ, accompanied by a reduction in postoperative complications [48], while other studies recommend not wasting time on two-stage benefits, and showed an increased rate of operative infectious complications after biliary drainage [49]. Other studies argue that preoperative drainage significantly improves the performance of free radical processes in the blood and does not influence long-term outcomes [50]. The most cautious researchers admit that an analysis of the literature does not confirm or refute the lack of alternatives to preoperative drainage of the biliary tract, noting the lengthening of hospital stay with this approach and the increase in associated cost, while providing low strength of evidence due to the low quality of the included trials [51]. Of course, this dissatisfaction with the available evidence is explained by real-life difficulties with the rapid collection of clinical data against the background of the rapid advancement of pharmacology, medical technology, and the ethical problems that arise in OJ cases. Therefore, this study was conducted using a retrospective analysis evaluating the results of treatment outcomes not only from a clinical but also from a pathophysiological standpoint.

The choice of antioxidant processes as an “arbitrator” in the issue of personalized approaches to treatment is explained by the fact that today most researchers share a common idea in which the progression toward liver and kidney failure that occurs in these patients is the main cause of death and is associated with free-radical processes, oxidative stress, leading to cellular energy deficiency [21,33,34,42,43,52,53,54]. 

The results of our study establish the feasibility of AOT including succinates, which is affirmed by the changes in the levels of markers of free radical processes and lipid peroxidation in patients with OJ. Further, the contribution of AOT to earlier stabilization of liver function is achieved by the normalization of laboratory parameters, an improvement in the clinical picture of the disease, a significant reduction in the number of complications by 2-fold from 23% to 11.5%, a reduction in the duration of preoperative drainage of the biliary tract by 1.5-fold, a reduction in the length of stay of patients in intensive care units. Our study recorded a decrease in hospital deaths by 4.5 times from 18% to 4% (perhaps this is due to a not very large sample size, but the positive result of using AOT is obvious, and the sample is representative and sufficient for reliable conclusions about the benefits of using AOT).

## 5. Conclusions

The pathogenesis of benign and, especially, malignant mammary glands is associated with impaired glycolysis, oxidative phosphorylation, and, as a result, oxidative stress with excessive lipid peroxidation.

The pathophysiology of OJ is associated with the mutually potentiating effects of hepatotoxicity, inflammation, lipid peroxidation, and glycolysis disorders leading to higher mortality. Early evaluation of prognostic markers of direct (and total) bilirubin levels, MDA, glycemia, and leukocytosis allows to predict outcomes on the day of hospitalization and favors the prompt adjustment of treatment.

The introduction of antioxidants into the complex treatment of patients will improve the immediate treatment results and ultimately reduce adverse outcomes.

## 6. Research Limitations

We understand that our work is not devoid of some shortcomings. As with any longitudinal clinical study, we always want a much larger number of observations to obtain the most reliable results. In addition, it would be interesting to obtain the comparative efficacy of different antioxidant drugs. We did not include in the study patients in extremely serious condition, with severe concomitant somatic pathology, as well as with metastasis, because multiple organ failure in such patients would reduce the effects of any therapy, including antioxidant therapy. We were forced to limit ourselves not only to the inclusion criteria but also to the period of material recruitment, as there is a constant change in recommended medicines and standards for the provision of medical care. In this case, the groups would not be comparable and the achievement of the goal set in the work would be impossible. We were forced to limit ourselves to 2020 due to the development of the pandemic and changes in treatment regimens. These limitations will be the basis for our subsequent work.

## Figures and Tables

**Table 1:** Distribution of patients by sex, age, duration, severity, severity, and etiology of OJ at admission.

	I Comparison Group (*n* = 61)	II Group AOT (*n* = 52)	*p*-Value	Total (*n* = 113)
Distribution of patients by sex				
Men	23 (37.7%)	24 (46.2%)	0.364	47 (41.6%)
Women	38 (62.3%)	28 (53.8%)		66 (58.4%)
Distribution by age, years				
≤50	10 (16.4%)	3 (5.8%)	0.158	13 (11.5%)
50–64	11 (18.0%)	11 (21.1%)		22 (19.5%)
65–79	21 (34.4%)	26 (50.0%)		47 (41.6%)
≥ 80	19 (31.3%)	12 (23.1%)		31 (27.4%)
Average age	67.2 ± 16.5 71 [57:81]	69.4 ± 14.6 75 [61:79]	0.556	68.2 ± 15.6 72 [58:80]
Distribution of patients by the severity of the condition (on the SOFA scale) at hospitalization				
<4 points (n;%)	33 (54.1%)	30 (57.7%)	0.709	63 (55.8%)
≥4 points (n;%)	28 (45.9%)	22 (42.3%)		50 (44.2%)
Distribution of patients according to the severity of jaundice upon admission (according to the level of total bilirubin, μmol/L)				
<100 μmol/L	22 (36.1%)	15 (28.8%)	0.284	37 (32.7%)
100–150 μmol/L	13 (21.3%)	18 (34.6%)		31 (27.4%)
>150 μmol/L	26 (42.6%)	19 (36.5%)		45 (39.8%)
Distribution of patients by the etiology of obstructive jaundice				
Neoplastic jaundice	18 (29.5%)	15 (28.8%)	0.939	33 (29.2%)
Non-neoplastic jaundice	43 (70.5%)	37 (71.2%)		80 (70.8%)
Including:			0.597	
Cholelithiasis	17 (27.9%)	17 (32.7%)		34 (30.1%)
Cholangitis	6 (9.8%)	8 (15.4%)		14 (12.4%)
Acute pancreatitis	5 (8.2%)	5 (9.6%)		10 (8.8%)
Distal choledoch stenosis	15 (24.6%)	7 (13.5%)		22 (19.5%)
Duration of clinical manifestations of jaundice at the time of hospitalization (self-reported and/or referred by a caregiver)				
0–2 days	15 (24.6%)	13 (25.0%)	0.673	28 (24.8%)
3–7 days	28 (45.9%)	20 (38.5%)		48 (42.5%)
>7 days	18 (29.5%)	19 (36.5%)		37 (32.7%)
Stay in intensive care units				
No	35 (57.4%)	32 (61.5%)	0.654	67 (59.3%)
Were	26 (42.6%)	20 (38.5%)		46 (40.7%)
Surgical treatments for obstructive jaundice				
Endoscopic papillo-sphincterotomy	27 (44.3%)	30 (57.7%)	0.560	57 (50.4%)
Nasobiliary drainage + stenting	12 (19.7%)	10 (19.2%)		22 (19.5%)
Cholecystostomy and cholecystectomy with drainage,	22 (36.1%)	12 (23.1%)		34 (30.1%)
Including:				
Microcholecystostomy/Gallbladder puncture	13 (21.3%)	4 (7.7%)		17 (15.0%)
Cholecystectomy + drainage	9 (14.8%)	8 (15.4%)		17 (15.0%)

The diameter of the common bile duct according to the ultrasound examination was also indistinguishable in both groups. In the comparison group, the average diameter of the common bile duct was 12 mm (interquartile range [IQR], 10–15 mm) and in the AOT group 14 mm (IQR, 11–17 mm) (*p* = 0.162).

**Table 2:** Comparison of hospital outcome and severity at admission of patients with obstructive jaundice (*p* = 0.002; Pearson’s X^2^).

Outcome	n, %	<4 Points on SOFA	≥4 Points on SOFA	Total
Survivors	n	61	39	100
	% Outcome	61.0%	39.0%	100%
	% SOFA	96.8%	78.0%	88.5%
Dead	n	2	11	13
	% Outcome	15.4%	84.6%	100%
	% SOFA	3.2%	22.0%	11.5%

**Table 3:** Dynamics of biochemical markers of liver function in patients with obstructive jaundice who received and did not receive antioxidant therapy.

Indicator	Day	Comparison Group (*n* = 61)		Antioxidant Therapy (*n* = 52)		*p*-Value	
Total bilirubin, μmol/L	0	129.7 73.8/193.1		129.0 94.5/189.0		0.641	
	3	47.4 23.0/152.9		41.5 21.9/101.2		0.257	
	8	46.0 21.1/78.0		25.8 13.7/63.9		**0.031**	
		Non-tumor	Tumor	Non-tumor	Tumor	Non-tumor	Tumor
	1	118 69/192	162 84.2/296	114 82/141	189 156.7/406	0.806	0.193
	3	29.2 21.2/58	112.6 73/224.3	30.4 21.1/55.7	136.1 80.4/165.1	0.519	0.354
	8	24.4 19.6/60.1	138.4 62.4/235	20.8 13.0/34.9	73.1 52.4/114.5	**0.047**	0.135
Direct bilirubin, μmol/L	0	90.3 50.5/147.0		81.5 49.7/149.5		0.915	
	3	44.1 14.7/75.7		19.7 9.9/35.7		**0.008**	
	8	25.8 12.5/71.0		10.5 7.0/23.1		**0.0001**	
		Non-tumor	Tumor	Non-tumor	Tumor	Non-tumor	Tumor
	1	87.9 49.8/109	134 51.5/228.2	76.1 47.0/107.0	163 87.7/351	0.493	0.181
	3	20.1 12.6/52.0	64.4 54.5/131.7	12.0 8.7/21.7	59.1 30.2/96.4	**0.005**	0.384
	8	18.3 8.3/30.6	68.4 46.4/147.3	8.3 6.6/12.8	23.9 18.8/43.4	**0.004**	**0.004**
Indirect bilirubin, μmol/L	0	32.6 19.0/83.3		34.1 21.5/60.6		0.807	
	3	25.4 11.3/73.4		28.6 13.6/43.6		0.980	
	8	22.0 13.7/35.3		18.6 12.0/39.3		0.520	
		Non-tumor	Tumor	Non-tumor	Tumor	Non-tumor	Tumor
	1	29.0 21.1/69.5	75.4 16/96.8	29.1 19.0/47.0	63.1 40.5/89	0.508	0.828
	3	15.8 7.4/29.5	82.6 29.1/149	17.4 9.7/31.2	57 38.4/80.8	0.647	0.384
	8	15.1 11.2/22.9	61.2 22.3/148.8	14.3 10.1/23.9	49.2 31.4/70.8	0.836	0.401
Alanine aminotransferase, U/L	1	189.2 103.3/293.8		193.4 118.9/261.2		0.951	
	3	68.3 51.0/130		71.2 52.5/140.3		0.914	
	8	68.3 41.9/94.5		48.6 31.1/75.9		**0.045**	
		Non-tumor	Tumor	Non-tumor	Tumor	Non-tumor	Tumor
	1	211.7 87.0/332.0	178.1 119.5/269.4	225.6 123.9/286.2	176.0 82.7/213	0.698	0.587
	3	80.1 55.4/139	62.3 45.4/122.5	64.8 54.4/144.6	77.6 45.3/131.3	0.961	0.955
	8	69.2 39.4/91.3	68.3 50.4/137.1	53.1 31.9/81.4	46.7 30.7/71.4	0.109	0.156
Aspartate aminotransferase, U/L	1	127 96.7/195.5		113 80.5/209.4		0.386	
	3	67 43.2/92		58 42.1/93.7		**0.002**	
	8	63.3 36/97		38.9 27.4/58.3		**0.013**	
		Non-tumor	Tumor	Non-tumor	Tumor	Non-tumor	Tumor
	1	121.8 94/195.4	132.9 102/170	119.0 85/219	88.9 59.7/146.5	0.954	0.097
	3	64.5 40/69	67.6 55/77.3	58 42/90.4	60.4 45.9/118.4	0.771	0.865
	8	65.6 34.6/85.2	63.3 44.8/116.2	36.9 29.9/53.7	43.3 26.4/60.7	**0.025**	**0.047**

Note: data are presented as median (first row); lower 25%/upper 75% quartile (second row).

**Table 4:** Dynamics of the number of blood leukocytes changes in patients with obstructive jaundice of tumor and non-tumor genesis, who received and did not receive antioxidant therapy.

Day	Comparison Group (*n* = 61)		Antioxidant Therapy (*n* = 52)		*p*-Value	
1	7.3 5.7/10.9		7.5 5.8/10.3		0.972	
3	7.6 5.8/11.2		6.7 5.0/8.4		**0.049**	
8	8.1 5.7/12.2		6.3 5.0/9.0		**0.022**	
	Non-tumor	Tumor	Non-tumor	Tumor	Non-tumor	Tumor
1	7.3 5.9/11.6	7.3 5.7/7.7	7.5 6.0/10.6	7.1 5.1/9.6	0.912	0.814
3	7.6 5.8/11.4	7.7 6.0/8.6	6.5 5.1/7.6	7.4 5.2/9.7	0.116	0.760
8	8.1 5.7/12.8	7.8 5.9/10.7	6.0 5.3/8.5	8.0 4.6/9.9	**0.030**	0.476

Note: data are presented as median (the first row); lower 25%/upper 75% quartile (the second row).

**Table 5:** Dynamics of lactate dehydrogenase and glucose changes in blood in patients with obstructive jaundice of tumor and non-tumor origin, among those who received and did not receive antioxidant therapy.

Indicator	Day	Comparison Group (*n* = 61)		Antioxidant Therapy (*n* = 52)		*p*-Value	
Lactate dehydrogenase (N: 130–230 U/L)	1	497 357/573		472 361/579		0.994	
	3	423 342/521		454 374/559		0.362	
	8	544 345/603		410 353/459		**0.046**	
		Non-tumor	Tumor	Non-tumor	Tumor	Non-tumor	Tumor
	1	438 352/615	528 451/542	468 377/577	474 349/586	0.803	0.872
	3	404 342/485	544 354/606	440 361/508	553 454/664	0.501	0.479
	8	435 340/589	574 367/615	411 363/452	405 333/428	0.457	**0.048**
Glucose (N: 2.3–6.0 mmol/L on an empty stomach)	1	5.4 4.5/6.3		5.9 5.0/7.2		0.160	
	3	5.9 5.3/7.0		6.0 5.2/7.1		0.756	
	8	6.2 4.7/8.6		5.85 5.4/6.5		0.639	
		Non-tumor	Tumor	Non-tumor	Tumor	Non-tumor	Tumor
	1	5.6 4.6/6.3	4.8 4.4/6.4	5.8 5.3/6.8	6.2 4.6/7.3	0.506	0.401
	3	5.7 4.8/6.3	6.6 5.6/11.3	5.7 5.0/6.7	7.3 6.3/7.7	0.770	0.654
	8	5.3 4.6/6.4	7.2 6.5/13.9	5.6 5.0/6.1	6.4 5.7/7.1	0.352	**0.037**

Note: Data are presented as median (the first row). lower 25%/upper 75% quartile (the second row).

**Table 6:** Hospital outcome of patients in the study groups.

Outcome	I Comparison Group (*n* =61)	II AOT Group (*n* = 52)	*p*-Value	Total (*n* = 113)
Discharged	50 (82.0%)	50 (96.2%)	0.018	100 (88.5%)
Dead	11 (18.0%)	2 (3.8%)		13 (11.5%)
Non-tumor OJ				
Discharged Dead	37 (86.0%) 6 (14.0%)	35 (94.6%) 2 (5.4%)	0.204	72 (90.0%) 8 (10.0%)
Tumor OJ				
Discharged Dead	13 (72.2%) 5 (27.8%)	15 (100.0%) 0	0.027	28 (84.8%) 5 (15.2%)

The data obtained support the feasibility and applicability of AOT as an important component of pathogenetic therapy when performing various types of surgical procedures in patients with OJ of various genesis and severity.

## Data Availability

Data is contained within the article.
